# SaLTy: a novel Staphylococcus aureus Lineage Typer

**DOI:** 10.1099/mgen.0.001250

**Published:** 2024-05-13

**Authors:** Liam Cheney, Michael Payne, Sandeep Kaur, Ruiting Lan

**Affiliations:** 1 School of Biotechnology and Biomolecular Science, University of New South Wales, Sydney, Australia

**Keywords:** genomic surveillance, genomic typing, microbial genomics, population structure, *Staphylococcus aureus*

## Abstract

*Staphylococcus aureus* asymptomatically colonises 30 % of humans but can also cause a range of diseases, which can be fatal. In 2017 *S*. *aureus* was associated with 20 000 deaths in the USA alone. Dividing *S. aureus* isolates into smaller sub-groups can reveal the emergence of distinct sub-populations with varying potential to cause infections. Despite multiple molecular typing methods categorising such sub-groups, they do not take full advantage of *S. aureus* genome sequences when describing the fundamental population structure of the species. In this study, we developed *Staphylococcus aureus* Lineage Typing (SaLTy), which rapidly divides the species into 61 phylogenetically congruent lineages. Alleles of three core genes were identified that uniquely define the 61 lineages and were used for SaLTy typing. SaLTy was validated on 5000 genomes and 99.12 % (4956/5000) of isolates were assigned the correct lineage. We compared SaLTy lineages to previously calculated clonal complexes (CCs) from BIGSdb (*n*=21 173). SALTy improves on CCs by grouping isolates congruently with phylogenetic structure. SaLTy lineages were further used to describe the carriage of Staphylococcal chromosomal cassette containing *mecA* (SCC*mec*) which is carried by methicillin-resistant *S. aureus* (MRSA). Most lineages had isolates lacking SCC*mec* and the four largest lineages varied in SCC*mec* over time. Classifying isolates into SaLTy lineages, which were further SCC*mec* typed, allowed SaLTy to describe high-level MRSA epidemiology. We provide SaLTy as a simple typing method that defines phylogenetic lineages (https://github.com/LanLab/SaLTy). SaLTy is highly accurate and can quickly analyse large amounts of *S. aureus* genome data. SaLTy will aid the characterisation of *S. aureus* populations and ongoing surveillance of sub-groups that threaten human health.

**Impact Statement**

*Staphylococcus aureus* is a highly diverse species consisting of large groups of related isolates. These groups can differ in virulence and antibiotic resistance. In this study we firstly used genomic data from 5000 isolates that are representative of the overall population to identify 61 lineages that contain the entire diversity of the species. We then created a tool (SaLTy) to assign new genomes to these lineages using the alleles of three specifically selected loci. SaLTy was used to assign lineages to 50 481 isolates. SaLTy assignments were consistent with the underlying phylogenetic relationships of the isolates. SaLTy lineages were also used to describe differences in the proportion and type of methicillin resistant *S. aureus* (MRSA) isolates in the population, enabling spatial and temporal analysis of MRSA lineages. SaLTy provides a simple tool to accurately and rapidly assign any genome to lineages that represent the population structure of the species.

## Data Summary

No new genome sequencing data was generated in this study. All genome sequencing data used in the study is publicly available through National Centre for Biotechnology Information, Sequence Read Archive and accessions have been included in supplementary materials. The authors confirm all remaining supporting data, code and protocols have been provided within the article or through supplementary data files.

## Introduction

*Staphylococcus aureus* is a bacterium of major public health concern that asymptomatically colonises ~30 % of the human population [1]. *S. aureus* causes many forms of disease and is a leading cause of bacteraemia and endocarditis [2]. Four waves of *S. aureus* antimicrobial resistance (AMR) emergence have been reported since the 1940s, further complicating the treatment of infections [3]. Over this time, *S. aureus* has evolved resistance to commonly prescribed antimicrobials, encompassing nearly all β-lactam antibiotics, including methicillin, leading to the emergence of methicillin-resistant *S. aureus* (MRSA) [4,6]. This resistance is caused by the *mecA* gene encoded on the Staphylococcal chromosomal cassette *mec* (SCC*mec*). Transmission of *S. aureus*, especially MRSA, was previously linked to healthcare-associated settings, however, in recent decades community-associated MRSA in settings with no direct clinical links has been widely reported [7,10].

Division of *S. aureus* into existing or emerging subpopulations is important for genomic surveillance. Additionally, some groups differ in their capacity to cause infection. Numerous molecular typing technologies have been developed to systematically classify these groups [11,13]. Pulsed-field gel electrophoresis (PFGE) was once considered the ‘gold standard’ of *S. aureus* classification. PFGE has been widely used to characterise *S. aureus* spread at the continent, country and local levels [14,17]. A limitation of PFGE is inconsistency across different laboratories due to variability in interpreting banding patterns [13]. The seven-gene multilocus sequence typing (MLST) method for *S. aureus* (developed in 2000), addressed this limitation by enabling standardised and unambiguous interlaboratory comparison of isolates[12]. Using this approach, the species can be subdivided into sequence types (STs), where isolates with the same ST contain identical alleles at seven loci. MLST has been applied to large collections of isolates and has defined both globally distributed and regionally restricted STs [18,19]. Closely related STs can also be grouped using clonal complexing [20]. A clonal complex (CC) groups STs that differ by a maximum of one allelic difference in the seven genes of the MLST scheme. The genetic similarity of diverse isolates has been described extensively using the CC nomenclature [21,23].

Whole genome sequencing (WGS) has been used to generate greater than 80 000 *S*. *aureus* genomes which can be used to classify the species population structure [24]. Capitalising on this rapidly growing WGS dataset, the conventional MLST method has been extended to include species core genes (core genome MLST, cgMLST) [25,27]. In 2014, an *S. aureus* cgMLST scheme was published that consisted of 1861 core loci [28]. The *S. aureus* cgMLST typing scheme thus generates allelic profiles using 1861 core loci, greatly increasing strain typing resolution compared with seven-gene MLST. The high resolution of cgMLST typing is suitable for distinguishing closely related isolates, such as identifying outbreak isolates transmitted within a hospital setting [29,30]. However, core genome STs (cgSTs) cannot describe the genetic similarity of diverse isolates since any new allele at any of the core loci will cause the assignment of a novel cgST. The allele profiles generated from cgMLST typing can instead be used to investigate the population structure between diverse isolates using clustering of cgSTs [31,32], in a manner similar to CCs. The development of clustering algorithms capable of processing large datasets has facilitated the identification of clusters from genomic data using cgMLST allele profiles or kmers, from thousands of isolates [27,33,35]. Clustering allele profiles has the potential to reconstruct the population structure of *S. aureus* by identifying the relationships of highly diverse isolates. To the authors’ knowledge, the clustering of cgMLST allele profiles has not previously been used to reconstruct the population structure of *S. aureus*.

In this study, we developed a novel tool that uses three core loci for the species-wide classification of *S. aureus* named *S. aureus* Lineage Typer (SaLTy). Developing SaLTy involved: (1) dividing the species into lineages by using clustered cgSTs, (2) selecting alleles of core genes that were specific to the lineages, (3) validating the selected core genes, and (4) designing the SaLTy algorithm. The usefulness of SaLTy was then investigated by dividing over 50 000 *S*. *aureus* genomes into lineages, demonstrating advantages over CCs, and characterising AMR trends within and between SaLTy lineages.

## Methods

### Dataset curation

Paired-end short read *S. aureus* WGS was downloaded from the NCBI Sequence Read Archive (SRA) using NCBI-genome-download on 15 March 2020 (v0.3.1) [36]. Contamination was identified with Kraken (v1.0) and read sets with more than 15 % of non-*S. aureus* reads were removed [37]. Raw reads were assembled by the pipeline developed for Multilevel genome typing [38]. Briefly, the procedure trimmed raw reads with Trimmomatic (v0.39), generated assemblies with SPAdes (v3.13.1), and calculated assembly quality metrics with QUAST (v5.1) [39,41]. Assemblies that met the following quality criteria were retained: (i) between 2.38 to 3.22 megabases, (ii) n50 greater than 50 000, (iii) average contig size (base pairs) greater than 15 000, (iv) fewer than 150 contigs, (v) and a GC% of between 32.5 and 33 %. Assemblies that passed these quality filters were assigned STs with mlst (v2.10) [42]. The entire set of filtered genomes formed the species dataset. A subset of 10 000 isolates was created including representatives of all STs and was named the species representative dataset as detailed in the results (Dataset S1, available online (https://figshare.com/articles/dataset/SaLTy_publication_supplementary_datasets/25632786)). This dataset was further divided into one selection dataset and one validation dataset each with 5000 isolates.

### Core genome validation

Each locus from a core genome of 1861 loci, published in 2014 [28], was assessed across the representative dataset and assigned to one of three categories. First, when greater than 20 % of the locus sequence was missing in greater than 1 % of isolates it was denoted as 'absent'. Second, when less than 20 % of the locus sequence was missing in greater than 1 % of isolates was denoted as 'partially missing'. All other loci were denoted as being present. The percentage of sequence present was determined by interrogating alleles called by the Allele Calling Pipeline of the Multilevel genome typing [38]. Selected settings for the Multilevel genome typing were a blast (v2.9) similarity of 80 % and a 16 bp sliding window.

### SaLTy design: lineage identification through hierarchical clustering

Alleles for the species representative dataset were called with the allele calling pipeline used in Multilevel Genome Typing [38]. A local cgMLST database was then set up to generate cgMLST profiles by processing the allele calls from each isolate. An in-house python script calculated the pairwise number of allele differences between all allele profiles (*Script 1*). Isolates were separated into clusters with an agglomerative approach (single-linkage clustering). This method functions by adding a given isolate to a cluster if it has fewer than a certain number of cgMLST allele differences (allele threshold) from any member of that cluster. Clusters were defined at a range of allele thresholds (0 to 1713 alleles) and the silhouette index (SI) for clustering at each allele threshold was calculated (*Script 1*). The phylogenetic relationship of isolates was visualised using GrapeTree (v1.5). GrapeTree was used to generate a neighbour joining phylogeny from allele profiles using the RapidNJ algorithm [43,44]. Isolates of the phylogeny were coloured by their assigned cluster at allele threshold 1026. PopPUNK was used to crosscheck the SaLTy lineages identified. PopPUNK clusters were called using the poppunk_assign command within PopPUNK (version 2.6.5) using the precomputed *S. aureus* reference database (version 1) [34].

### SaLTy design: selection of a three gene combination for lineage assignment

A definition dataset comprising 5000 isolates (randomly chosen from the 10 000 isolates in the species representative dataset) was queried using an in-house python script (*Script 2*) to identify cluster-specific alleles in core genes. Using a gene-by-gene approach, an allele was assigned to one of the clusters when present in at least 50 % of isolates in that cluster and uniquely carried by isolates of that cluster. The following three performance metrics were calculated for every cluster: accuracy, specificity, and sensitivity (Methods S1.1). For all clusters, each of the accuracy, specificity, and sensitivity metrics were averaged to create a macro-accuracy, macro-specificity, and macro-sensitivity respectively. Macro-statistics distributions were visualised in Prism (v9.3.1) [45]. Cluster-specific alleles were further selected from two and three gene combinations where any of the genes could be used to assign a lineage to increase macro-accuracy. Single genes with a macro-accuracy in the top 10 % were tested in two and three gene combinations. A single three gene combination was selected using a combination of five criteria (Methods S1.2, Dataset S2). These criteria were: (i) greater than 99 % accuracy; (ii) 100 % specificity; (iii) greater than 100 kbp distance between the three genes; (iv) less than 1 % isolates with none of the three specific alleles for a given lineage; and (v) loci with the fewest isolates belonging lineages where only one of the three genes had a specific allele. Each metric progressively filtered three gene combinations until a single three gene combination passed the filtering thresholds.

### SaLTy design: validation of lineage assignment using the selected three gene combination

The validation dataset comprised the remaining half of the species representative dataset and was independent of the definition dataset. For each isolate, the alleles for the selected three gene combination were extracted from the previously generated allele profiles (Section SaLTy design: species division through hierarchical clustering). Allele calls for each of the three genes in the combination were used to determine the cluster of each isolate by checking the lineages that each allele was specifically found in (Dataset S3). The performance of the selected three gene combination was evaluated by comparing the cluster assigned to an isolate based on allele presence in one or more of those genes to the cluster assigned by hierarchal clustering of the genome of that isolate (Methods S1.2, Scripts 3–4). The performance of the three gene combination that was selected was examined using the validation dataset and the five criteria mentioned in the selection section.

### SaLTy algorithm development and application

The SaLTy pipeline was developed in python 3 (https://github.com/LanLab/SaLTy). Isolates were screened for exact matches to alleles in the three genes using KMA (v1.3.24) [46]. The SaLTy pipeline was developed such that it, (1) screens isolates against a KMA database of DNA sequences for the cluster specific alleles, (2) filters matches that are 100 % in both coverage and identity, and (3) assigns a lineage through cross-referencing the exact allele match with a table of alleles and specific clusters. An isolate is not assigned a lineage (i.e. is untypable) when it lacks an allele from all three loci, or carries a novel allele for all three loci.

The SaLTy pipeline was used to analyse the entire species dataset (Dataset S1). Lineage size distribution was visualised with Prism (v9.3.1) [45]. A timing function was built into the SaLTy pipeline for testing performance. The seconds required to assign a lineage for each isolate and the total time required to analyse multiple isolates was recorded (Dataset S1) using a single thread and 64 megabytes of memory.

### SaLTy and CC division of *S. aureus* STs from PubMLST

The PubMLST database had 21 883 *S*. *aureus* genome submissions with associated CC metadata as of 4 April 2020, which were downloaded and typed using the SaLTy pipeline. Tableau (v9.1) was used to visualise the grouping of the PubMLST isolates into SaLTy lineages and CCs [47]. A subset of 189 isolates from the PubMLST database, representing all SaLTy lineages for which a CC type was assigned, was generated. We included all isolates from a SaLTy lineage if that lineage had fewer than ten isolates assigned to it, otherwise ten isolates were randomly selected. Snippy (v4.6.0) [48] was used to call single nucleotide polymorphisms (SNPs) in the subset. Snippy called SNPs against the reference genome *S. aureus* COL GCA_000012045 which was selected because it was a closed genome that carried *mecA* [49]. SNPs predicted to be affected by recombination were removed with RecDetect (v6.1) [50]. A maximum likelihood phylogeny with 10 000 ultrafast bootstraps was generated with IqTree (v2.0.4) [44]. Branches of the phylogeny were coloured by bootstrap value, and, the SaLTy lineage and CC metadata labels were visualised with the Interactive Tool of Life (iTOL) [51].

### *In silico* SCCmec typing

The SCC*mec* type for an isolate was predicted *in silico* using Staphopia SCC*mec* [52] (Dataset S1). It should be noted that the publicly available version of Staphopia used in this study can only identify SCCmec types I–VII and recently described types (VIII–XIV) were not identifiable. The distribution of SCC*mec* types for isolates of each SaLTy lineage was visualised with Tableau (v9.1) [47].

## Results

### Curation of the species dataset and selection of representative datasets

Developing a *S. aureus* lineage typer required a high-quality genomic dataset. Initially, a species dataset (*n*=50 481) of quality filtered genomes was created (Dataset S1). The representative dataset was divided into 1665 conventional MLST types (Results S2.1, Fig. S1). A species representative dataset (*n*=10 000) was selected that sampled each MLST ST in proportion to the frequency of the MLST STs in the species dataset. The species representative dataset was further divided into a definition dataset (*n*=5000) and a validation dataset (*n*=5000). The definition and validation datasets were used to select and validate the core genes used in the SaLTy pipeline.

### Validation of the *S. aureus* cgMLST scheme using the representative dataset

An existing cgMLST scheme was evaluated for performance of core genes across the species [28]. The cgMLST scheme had 92 % (1713/1861) loci present in greater than or equal to 99 % of the representative dataset (Results S2.2, Fig. S2). Consequently a core genome of 1713 genes was used for further analysis.

### *S. aureus* population divided into 61 lineages

**Fig. 1.**
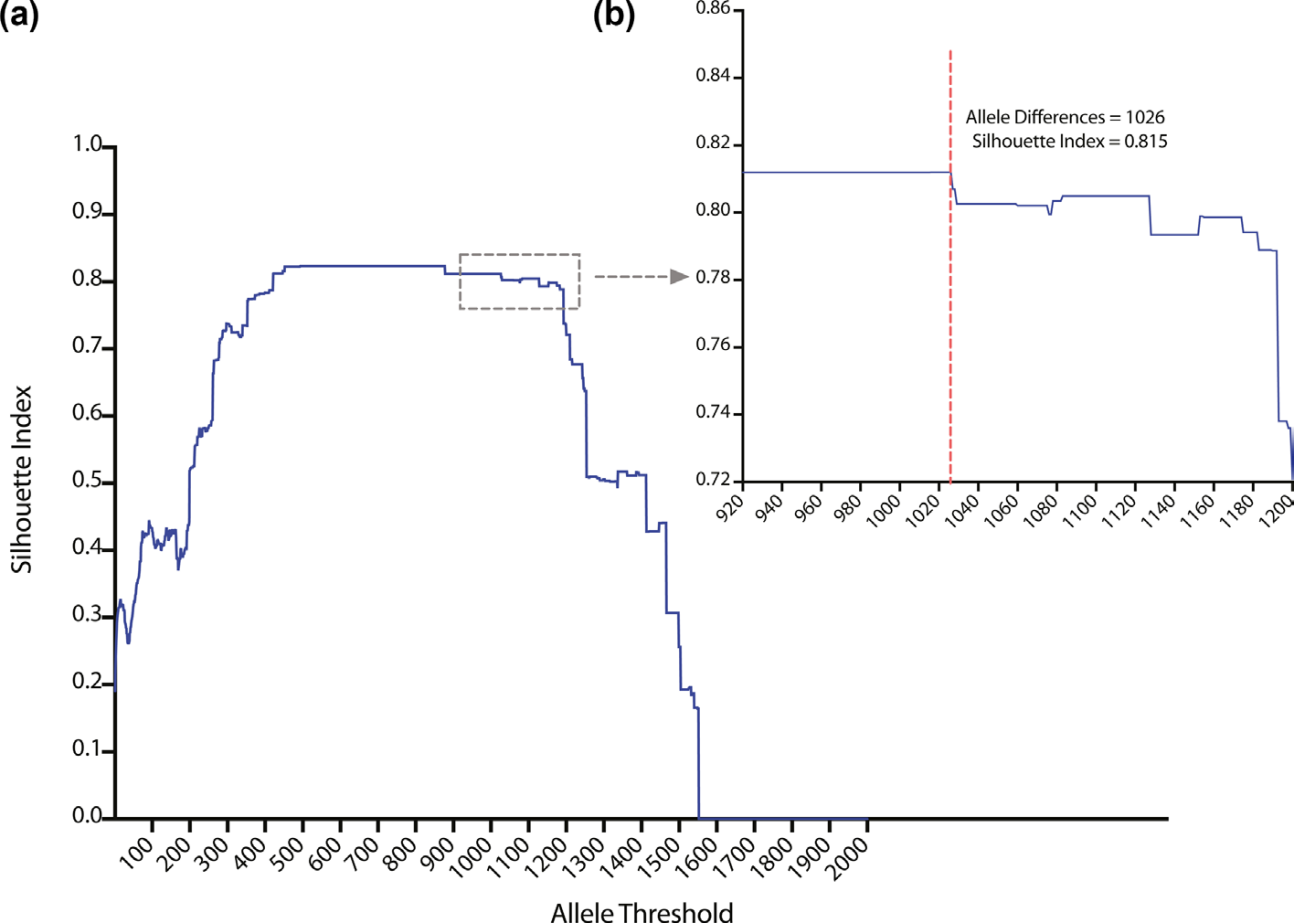
Silhouette indexing supports an allele threshold of 1026 to divide the species. The species representative dataset (*n*=10 000) was divided into clusters using hierarchal clustering with cut-offs from zero to 1713 allelic differences. The silhouette index for each allelic threshold is shown in blue. The dashed red line marks the allele threshold selected to cluster the dataset. Visualised in Prism v9.3.1 [45].

**Fig. 2.**
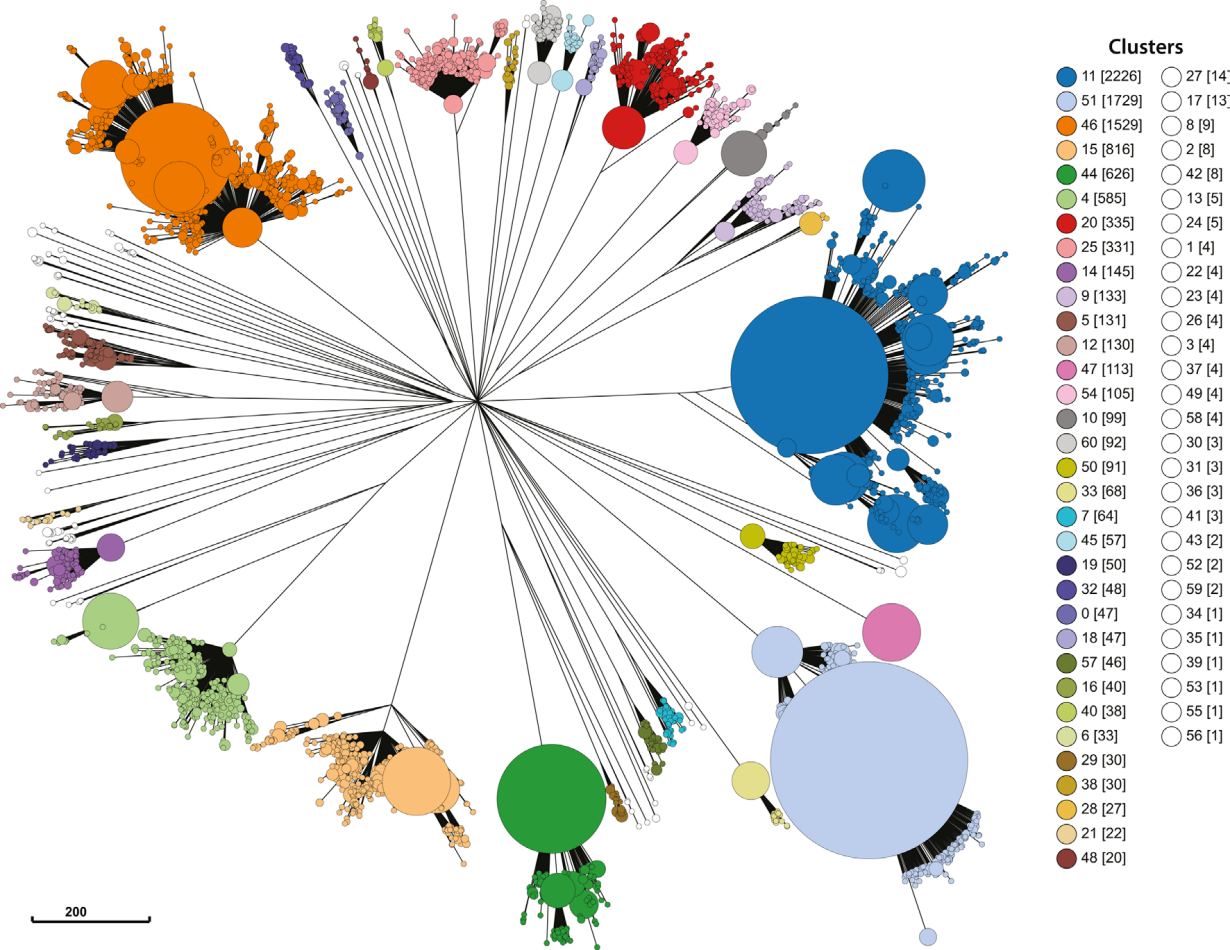
Hierarchal clusters with a 1026 allele difference cut-off are congruent with the species phylogenetic structure. The phylogenetic structure of the species representative dataset (*n*=10 000) was compared to 61 hierarchal clusters. Phylogenetic relationships were calculated from cgMLST profiles using the Rapid Neighbour Joining algorithm. Isolates were coloured by cluster type. The number of isolates assigned to each cluster was shown in square brackets. Clusters with <20 isolates were coloured white. Nodes were merged where branch lengths were less than 30 for clarity. The phylogeny was visualised in GrapeTree [44].

In order to identify subpopulations within the *S. aureus* species we clustered cgMLST allele profiles at thresholds from 0 to 1713 allele differences. Thresholds were evaluated for hierarchical clustering performance using the Silhouette Index (SI) (Fig. 1). An allele threshold of 1026 was chosen with an SI of 0.815 which was within the top 1.5 % of all SIs and indicated clusters were well separated. An allele difference of 493 had the highest SI (0.824), however, we chose an allele threshold of 1026 as that would cluster isolates into the more genetically divergent lineages and result in more stable clustering (Fig. 1). The population was divided into 61 clusters at the selected threshold of 1026 alleles. The five largest clusters (11, 51, 46, 15 and 44) cumulatively contained 69.25 % (6925/10 000) of the species representative dataset (Fig. S3A) and almost half of the clusters (49.18 %, 30/61) comprised <10 isolates (Fig. S3B). Visual inspection of the species phylogeny showed that all 61 clusters were concordant with distinct phylogenetic lineages, justifying the description of the clusters as lineages (Fig. 2).

### Selection of three genes with alleles specific to the 61 lineages

**Fig. 3.**
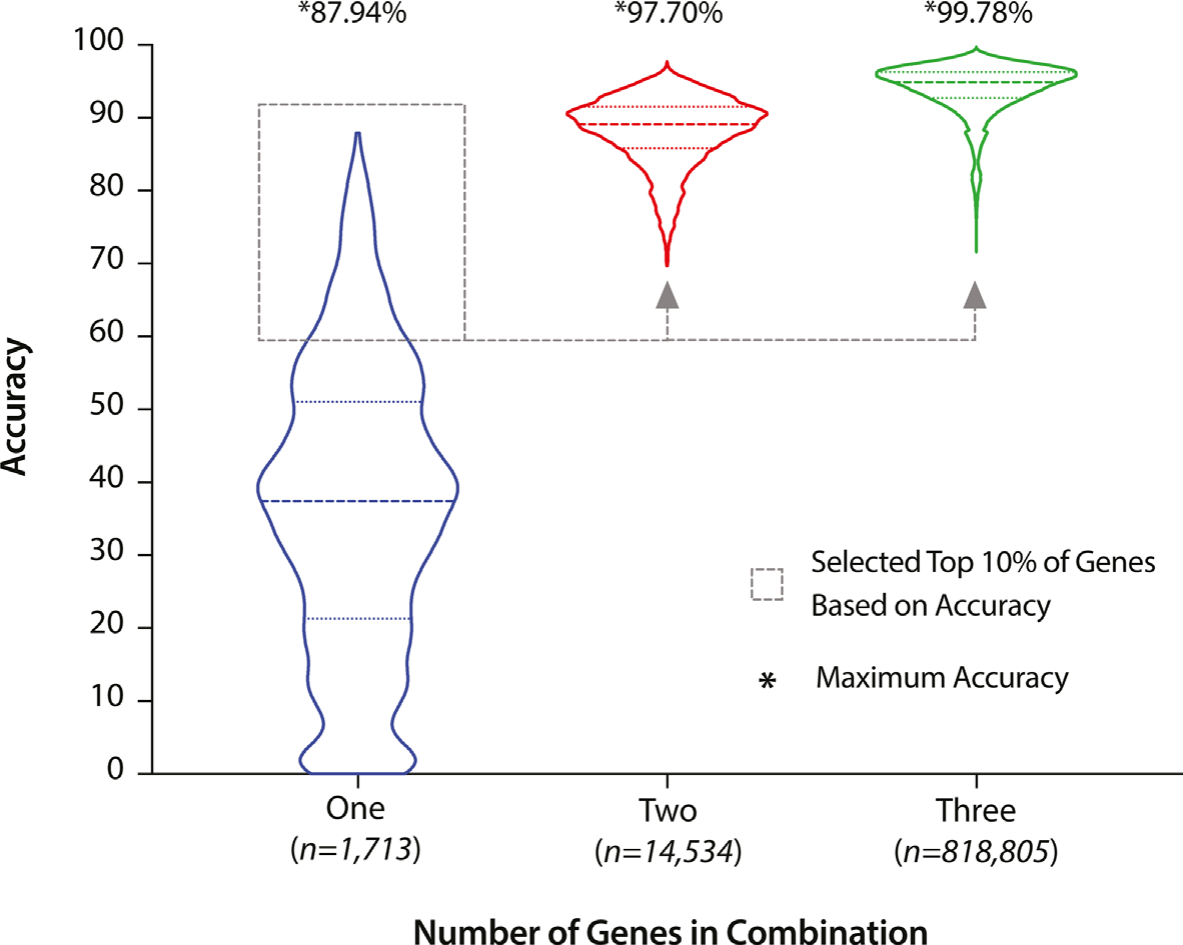
Increasing the number of genes used for specific alleles increased the accuracy of assignment to the 61 hierarchal clusters. Accuracy of one, two and three core gene combination in grouping isolates into the 61 hierarchal clusters was visualised. Initially, genes were identified that contained one distinct allele with more than 50 % sensitivity and 100 % specificity for every hierarchal cluster. The accuracy for each core gene in assigning a cluster using one allele for each cluster was shown in blue. Based on this accuracy, the top performing 10 % individual genes were selected to create two and three gene combinations (shown by grey dashed line). The number of two and three gene combinations was shown below the X-axis. The accuracy for two and three gene combinations was calculated by testing for the presence of an allele that is specific for a cluster in any of the genes in a combination. The accuracy for two and three gene combinations was shown in red and green respectively. The maximum accuracy for one, two and three gene combinations was labelled and marked by an asterisk.

While the assignment of an isolate to these 61 lineages is potentially useful, each addition requires running cgMLST and clustering which require significant time and computation. To solve this problem, we identified core genes whose alleles could be accurately used to assign the 61 defined lineages using half of the species representative dataset (termed the detection dataset, Results S2.3, Dataset S2). We compared the lineage assignment accuracy of alleles from a single gene, and, combinations of two and three genes (Fig. 3). Alleles from single, two and three gene combinations had maximum accuracies of 87.94, 97.90 and 99.78 % respectively (Fig. 3, marked by asterisks). Thus, selecting alleles from a three gene combination provided higher accuracy than selecting alleles from a two gene combination or a single marker gene. Each three-gene combination was further assessed based on five iterative metrics; using this approach, the best three-gene combination was SACOL0451-SACOL1908-SACOL2725 (Table S1, Dataset S2). The SaLTy tool was developed to assign an isolate to one of these 61 lineages using specific alleles for each lineage in one or more of the three genes (henceforth described as SaLTy lineages, Dataset S3).

### Independent validation of SACOL0451-SACOL1908-SACOL2725 within SaLTy

To validate the selection of the three genes, we examined the remaining 5000 isolates in the species representative set (termed the validation dataset). These isolates were not included in the selection of the SACOL0451-SACOL1908-SACOL2725 combination. Using these three genes, SaLTy was able to assign 4978 of the 5000 isolates (99.56 %) of the validation dataset to a lineage with only 22 isolates unassignable (Dataset S1). These 22 isolates in the validation dataset contained alleles in all three loci that were different from the lineage assignment alleles derived from the selection dataset and hence could not be assigned a lineage. For the successfully assigned isolates, the lineage assignment is identical to the single linkage clusters they were assigned to with allele threshold (1026) as the definition dataset. Additionally 98.07 % of lineage assignments were based on allele calls of two or three of the three genes.

### *S. aureus* population structure as described by SaLTy lineages

**Fig. 4.**
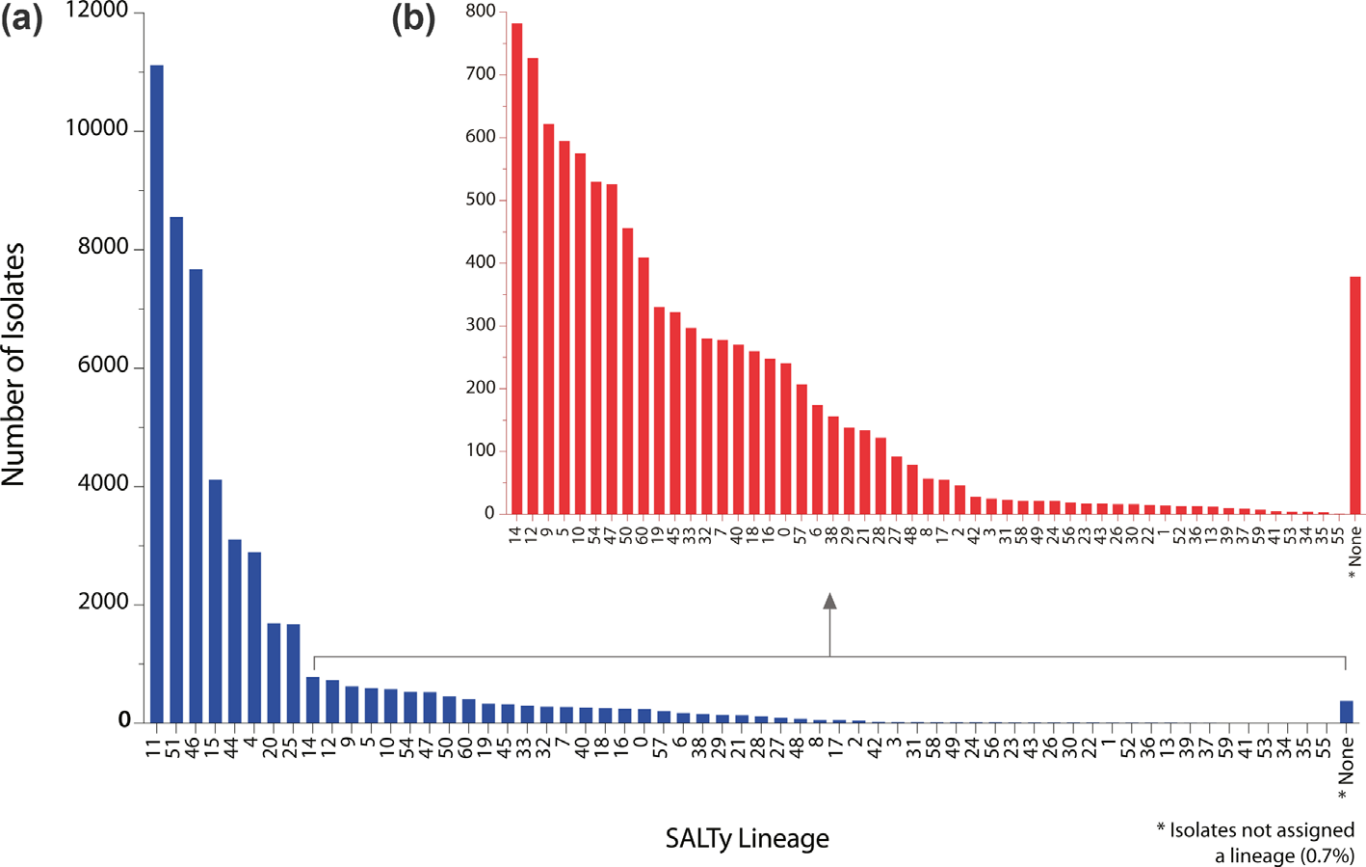
SaLTy divided the *S. aureus* species into 61 lineages. SaLTy processed and assigned a lineage to 99.3 % of the complete species dataset (*n*=50 481). Isolates not assigned a lineage were represented in the ‘None’ category (marked by asterisk). (**a**) The distribution of the sizes of all lineages across the dataset. (**b**) A subplot of cluster sizes that were assigned to <800 isolates. Visualised in Prism v9.3.1 [45].

The three validated genes, SACOL0451-SACOL1908-SACOL2725, applied within SaLTy allow lineage assignment without calling cgMLST types or hierarchal clustering. SaLTy was used to divide the species dataset (*n*=50 481) into lineages (Dataset S1). SaLTy successfully assigned a lineage to 99.25 % (50 100/50 481) of the isolates (Fig. 4). The three largest lineages were 11, 51 and 46, which included 54 % (27 335/50 481) of the genomes (Fig. 4a). There were seven lineages with ten or fewer isolates, and lineage 55 was the only singleton (Fig. 4b). The time required to assign an isolate to a lineage and the total time required to analyse the entire species dataset was recorded (Dataset S4).

### Computational performance of SaLTy

In the species dataset (*n*=50 481) an isolate was assigned a SaLTy lineage in an average of 0.133 s, and the entire dataset was analysed in 112 min and 3 s with one thread (Dataset S1).

### SaLTy lineage typing has a higher resolution than clonal complexing and SaLTy lineages are congruent with the maximum likelihood phylogeny and PopPUNK clusters

The concordance of the *S. aureus* clonal complexes and SaLTy lineages was investigated. The PubMLST database contained 21 173 *S. aureus* genomes with CCs assigned and were classified into ten CCs [53]. Using SaLTy, these isolates were assigned to 21 lineages. Comparing the SaLTy lineages to CCs showed that 50 % (5/10) of CCs were split into multiple SaLTy lineages (Fig. S4). SaLTy split CC1, CC5, CC8, CC97 and CC121, into three or more lineages each. Isolates in the remaining CCs, CC15, CC22, CC30, CC45 and CC93, were respectively typed as lineages 25, 51, 15, 4 and 47.

**Fig. 5.**
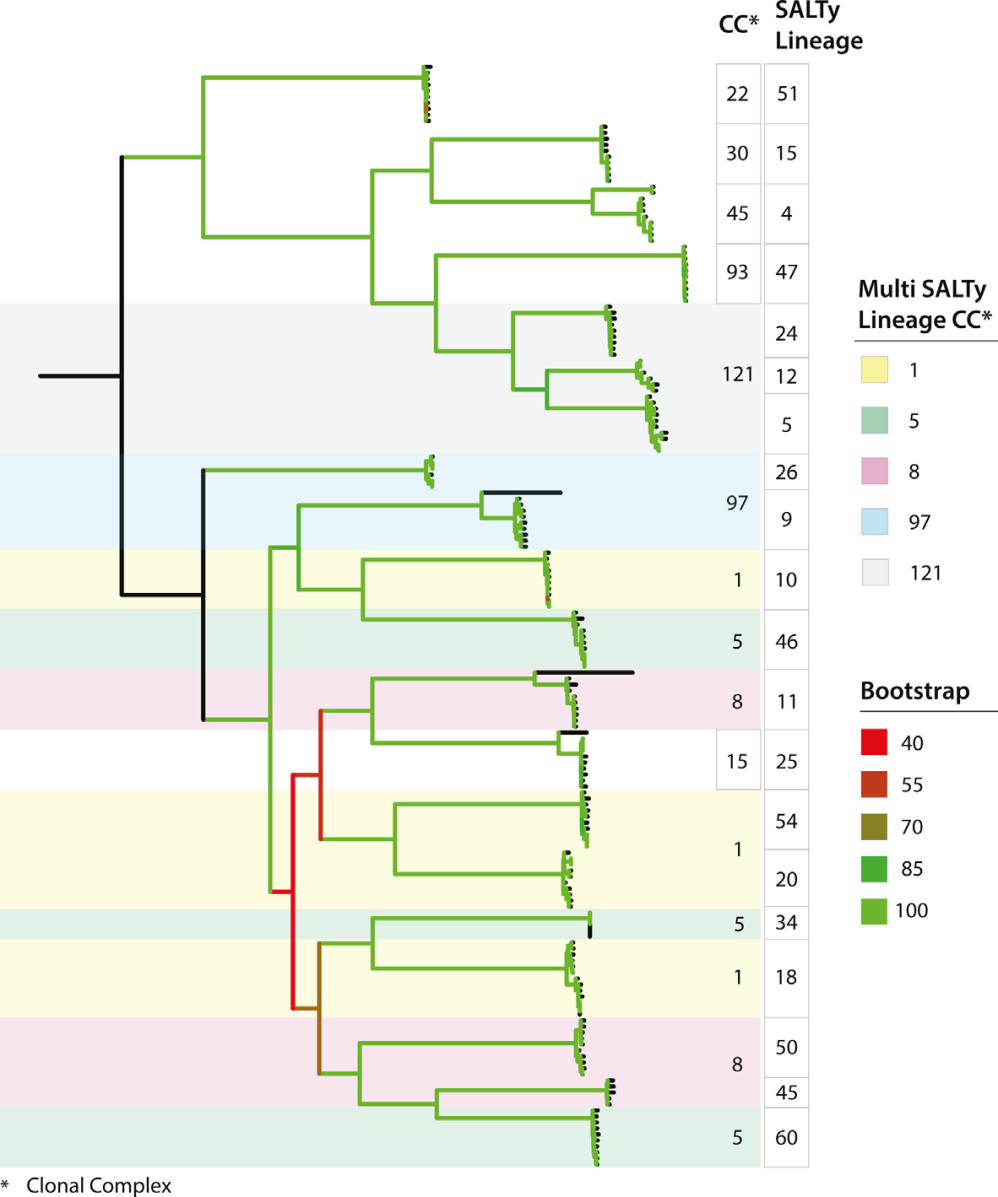
SaLTy splits clonal complexes (CCs) into multiple lineages that are congruent with the phylogeny. A maximum-likelihood phylogeny of *S. aureus* isolates (*n*=185) from multiple SaLTy lineages and CCs. The phylogeny was based on 33 275 non-recombinant SNPs. SaLTy lineages and CCs were annotated alongside. CCs splitting into multiple SaLTy lineages were coloured (shown in yellow, green, red, blue and grey). Branches were coloured by ultra-fast bootstrap values (*n*=10 000). Phylogeny was generated using IQ-TREE v2.0.4 and visualised in iTOL [51,79].

The groups of isolates defined by SaLTy and CC were compared by overlaying their assignments onto the maximum likelihood phylogeny in Fig. 5. The phylogeny included representatives from each of the 21 SaLTy lineages and ten CCs. SaLTy assigned lineages were congruent with lineages of the phylogeny and all 21 SaLTy lineages were monophyletic. By contrast, five of the ten CCs (CC1, CC5, CC8, CC97 and CC121) were polyphyletic, spanning multiple phylogenetic clades (Fig. 5). For example, CC1 included isolates from four clades which were individually assigned to lineages 10, 18, 20 and 54 by SaLTy (Fig. 5).

PopPUNK was further used to validate the SaLTy lineages. PopPUNK clusters were called using the species dataset (Dataset S1, *N*=50 481). Agreement between SaLTy lineages and PopPUNK clusters was very high with an adjusted rand score of 0.943 and a Normalized Mutual Information score of 0.967. Of the 61 SaLTy lineages, nine are split into two or more PopPunk clusters (total number of isolates in inconsistent clusters was 581). Of the 60 PopPUNK clusters one was split into two SaLTy lineages of 1671 and 7664 isolates.

### SaLTy lineages are heterogeneous in SCC*mec* type and the largest SaLTy lineages vary in SCC*mec* type over time

To describe the grouping of MRSA isolates and isolates lacking SCC*mec* (methicillin susceptible *S. aureus* (MSSA)), we predicted the SCC*mec* type for isolates in each SaLTy lineage (*n*=50 100). Isolates with a predicted SCC*mec* element were assumed to be MRSA, and isolates without a predicted SCC*mec* were assumed to be methicillin susceptible (MSSA). At least one isolate was predicted to carry SCC*mec* in 26 lineages (42.6 %, 26/61) (Dataset S1, Table S2). Five different SCC*mec* types (SCC*mec* I, II, III, IV and V or VII) were predicted in the 50 100 isolates in these 26 SaLTy lineages (Fig. S5). There were 14 lineages with two or more SCC*mec* types and 12 lineages with only a single SCC*mec* type. The lineages with multiple SCC*mec* types were considerably larger than lineages with a single SCC*mec* type. The largest lineage with multiple SCC*mec* types was lineage 11, which included 11 119 isolates, and the largest lineage with a single SCC*mec* type was lineage five, which included 590 isolates (Fig. S5A). The 35 remaining lineages had no isolates with a predicted SCC*mec* type (Fig. S5). Cumulatively, these SCC*mec* negative lineages contained 4543 isolates and were generally smaller than lineages with isolates carrying a SCC*mec* element. Lineage 25 (*n*=1671) was the largest lineage to have no isolate carrying an SCC*mec* element (Fig. S5A).

**Fig. 6.**
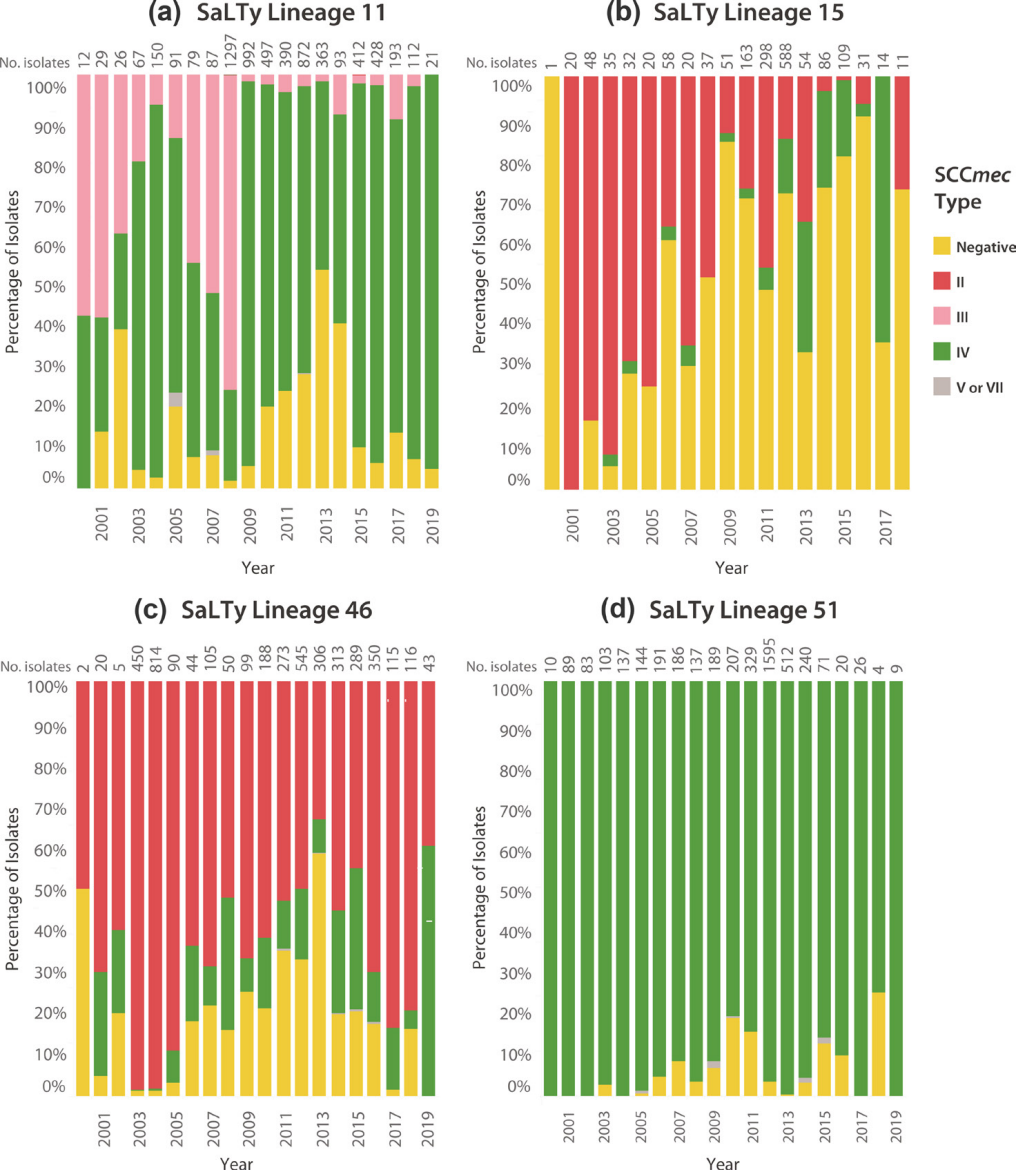
Comparison of the SCC*mec* types over time in SaLTy lineages 11, 15, 46 and 51. The SCC*mec* types in SaLTy lineages 11, 15, 46 and 51 between 2000 to 2019 were compared. SCC*mec* negative isolates were yellow, and SCC*mec* II, III, IV and V and VII were coloured as red, pink, green and grey respectively. The frequencies of predicted SCC*mec* types were shown as the percentage of the total isolates per year. The total number of isolates sampled from each year was shown above each column.

The changing frequencies of SCC*mec* types over time from 2001 to 2019 in the four largest SaLTy lineages were visualised (Fig. 6). Lineages 11, 15 and 46 all had a mixture of SCC*mec* types in almost all years sampled. Lineage 11 had both SCC*mec* III and IV between 2000 to 2008, and from 2009 onwards, the majority of isolates were SCC*mec* IV (Fig. 6a). Lineage 15 was predominately SCC*mec* II before 2012, but post-2012, was a combination of SCC*mec* II and IV (Fig. 6b). Additionally, lineage 15 was the only lineage to increase in the number of SCC*mec* negative isolates over time. Lineage 46 was a mixture of SCC*mec* II and IV each year, and within that mixture SCC*mec* II was more common for all years (except 2019) (Fig. 6c). Finally, lineage 51 was almost homogeneous in SCC*mec* type with SCC*mec* IV being the only type with the exception of a small number of SCC*mec* V or VII (Fig. 6d).

## Discussion

### SaLTy offers rapid whole species classification

WGS has ushered in a new era of *S. aureus* genomic epidemiology. *S. aureus* WGS is growing in popularity and will likely become routine for species-wide surveillance [54,55]. The majority of *S. aureus* WGS data has been generated in the past decade, which offers a new opportunity to develop rapid and scalable WGS-based classification technologies [56]. In this study, we developed a novel genomic typing technology named SaLTy. SaLTy is a rapid genome typer that divides the *S. aureus* species into groups consistent with phylogenetic lineages. The typing resolution of SaLTy is lower than traditional MLST and comparable to that of clonal complexing (Fig. 5) [12,28]. Unlike MLST and clonal complexing, the SaLTy lineages were defined through the high-resolution investigation of thousands of cgMLST allele profiles, and the lineages represent the fundamental population structure of the species (Fig. 2).

The population structure of *S. aureus* as a whole is most often studied through phylogenetics [57,59]. Databases have been developed that can build phylogenies with tens of thousands of isolates and generate interactive visualisations that aid in investigating genomic epidemiology [27,44]. However, incorporating newly sequenced *S. aureus* WGS data into a phylogeny requires rerunning the entire analysis. This makes phylogenetic reconstruction unfeasible for the ongoing interpretation of large numbers of newly sequenced *S. aureus* isolates. Therefore, there is a lack of genomic classification technologies that can rapidly assign lineages using *S. aureus* WGS.

Methods such as popPUNK, hierCC, SNP-address and LIN typing can assign large scale clusters to isolates but require the genomic comparison of the query isolate to large databases [33,35,60]. SaLTy can assign an isolate to the pre-defined SaLTy lineages by screening for alleles from three pre-selected core genes without analysing the remainder of the genome. SaLTy has been used to process over 50 000 *S*. *aureus* genomes and can be used to process future *S. aureus* WGS data (Fig. 3). We provide SaLTy as a freely available tool (https://github.com/LanLab/SaLTy) to facilitate ongoing surveillance of existing and emerging *S. aureus* lineages.

### SaLTy lineages describe MRSA and MSSA epidemiology

*S. aureus* is notorious for its ability to acquire genetic elements that confer resistance to antibiotics [61,64]. Traditional typing technologies have defined multiple sub-groups that have acquired AMR elements and proceeded to expand globally [22, 65,67]. Given that SaLTy defines lineages at the largest scale it is unlikely that one lineage will correspond to one of these genetic element acquisitions. Rather SaLTy can be used to examine the gain and loss of these elements in the context of the broader population structure of the species. SaLTy was used to separate the species into lineages of diverse isolates and show that over half the lineages (36/61) contained MRSA isolates (Figs 6 and S5). The genetic basis of MRSA is the presence of SCC*mec* which can be horizontally transferred between isolates. Variants of SCC*mec* have also been characterised that contribute to varying MRSA resistance profiles [68]. In one landmark study, the emergence of novel SCC*mec* types was used to define multiple waves of MRSA endemic spread [3]. We compared the changing frequencies of SCC*mec* types over time and showed the four largest SaLTy lineages varied in the carriage of SCC*mec* type. By describing the trends in SCC*mec* types in SaLTy lineages, we could show that large SaLTy lineages did not always carry the same SCC*mec* types, which is relevant when tracking common antibiotic resistance phenotypes.

SaLTy lineages will also be useful for tracking the gain of mobile elements and resistance. There were 35 SaLTy lineages containing only MSSA isolates. Classifying lineages of MSSA is important as MSSA isolates can acquire the SCC*mec* and become MRSA [69]. This phenomenon is not rare and the emergence of MRSA in the species has been shown to have independently occurred multiple times [4,70]. The acquisition of SCC*mec* can lead to rapid expansion of a lineage and SaLTy will be able to identify such lineages rapidly and accurately.

MSSA lineages that remain SCC*mec*-free are also essential to classify. MSSA is the largest cause of *S. aureus* related disease(s) and recent reports have found a resurgence in MSSA infections and a reduction in MRSA infections since the 2000s [71,74]. The combination of rapid SaLTy and SCC*mec* typing from genomic data should allow effective high-level MRSA and MSSA epidemiology and surveillance.

### SaLTy lineages represent the basic population structure of the species

A detailed understanding of the large-scale population structure of the species is the basis for investigating the evolution of both well-established and emerging subgroups [33,75]. The SaLTy lineages describe the large-scale population structures of *S. aureus* by separating greater than 99 % of *S. aureus* isolates into 61 lineages which represent ancestral stages of evolution (Fig. 2). As more genomes are sequenced, there may be isolates that are not within the 61 lineages. These new lineages can be defined and incorporated into SaLTy while maintaining existing definitions.

The *S. aureus* fundamental population structure has been analysed with clonal complexing [12, 22]. We compared clonal complexing and SaLTy division of the species and showed that only half of the CCs (5/10) were assigned a single SaLTy lineage (Fig. 5). Further investigation of the remaining CCs showed SaLTy divided these CCs into lineages consistent with the underlying phylogenetic relationships. A limitation of clonal complexing is that CCs are based on alleles from the seven gene MLST scheme that do not always reflect phylogenetic structure [20]. Another limitation of CCs is that they are only assigned to a subset of seven gene MLST STs. By contrast, SaLTy is able to describe the entire species including 40 lineages that had not been described using CCs.

SaLTy lineages were also compared to PopPUNK clusters and showed very high concordance including the division of multiple CCs into smaller lineages. PopPUNK uses kmer based comparison of the whole genome and so represents a methodologically independent measure of population structure [34]. SaLTy lineages were congruent with both PopPUNK clusters and the phylogenetic structure of the species providing a complete representation of the species population structure.

### The future extension of SaLTy in a PCR assay

SaLTy was initially developed to process *S. aureus* WGS data, however the cost of WGS could act as a barrier to its adoption. SaLTy could also be developed into a PCR-based amplicon typing technology [76,78]. The alleles from the three SaLTy genes could be used through (1) designing three simplex PCR assays or a single triplex PCR assay, (2) amplicon sequencing the PCR products, and (3) assigning a lineage through processing the amplicon sequencing with existing SaLTy genomics pipeline. Developing SaLTy into a laboratory-based method with amplicon sequencing that can be highly multiplexed would facilitate faster and larger scale lineage typing of *S. aureus* isolates without the need for WGS and large scale surveillance of *S. aureus* lineages in one health, environment and public health settings.

## Conclusion

In this study, we developed a novel genomic typing technology for *S. aureus* named SaLTy. SaLTy was used to resolve the high-level population structure of the species into 61 lineages and can rapidly assign these lineages to large volumes of *S. aureus* WGS data. The SaLTy lineages were used to describe the carriage of SCC*mec*. The four largest SaLTy lineages carried different frequencies of SCC*mec* variants over time while the majority of SaLTy lineages were MSSA. We anticipate SaLTy will facilitate the identification and ongoing surveillance of existing and emerging *S. aureus* lineages that poses a threat to human health.

## supplementary material

10.1099/mgen.0.001250 Supplementary Material 1.

## Abbreviations

AMR: antimicrobial resistance
CC: clonal complex
cgMLST: core genome multilocus sequence typing
MLST: multilocus sequence typing
MRSA: methicillin-resistant *S. aureus*
MSSA: methicillin susceptible *S. aureus*
PFGE: pulsed-field gel electrophoresis
SaLTy: *Staphylococcus aureus* lineage typing
SCCmec: Staphylococcal chromosomal cassette containing mecA
ST: sequence type
WGS: whole genome sequencing
