# Supplementary material for: SaLTy: a novel Staphylococcus aureus Lineage Typer
Source: Microb Genom. 2024 May 13;10(5):001250. doi: 10.1099/mgen.0.001250 (PMC11165655; doi:10.1099/mgen.0.001250)
Supplement: Supplementary Material 1. [file mgen-10-01250-s001.pdf]

1 Supplementary Methods

1.1 Calculating performance metrics for cluster specific alleles.

On a gene-by-gene basis, alleles were assigned as a marker (referred to as assigned alleles) for a single linkage cluster (SLC) (**Script 2**). For each cluster a confusion matrix was calculated by screening the assigned allele in the species representative dataset (*n=10,000*). The clusters defined in single linkage clustering were treated as the ground truth. The confusion matrix included the following: true positives (TP), false positives (FP), true negatives (TN), and false negatives (FN). A TP isolate carried the assigned allele, and the inferred cluster from the allele was the same as the cluster from SLC. A FP isolate carried the assigned allele, however, the inferred cluster from the allele was not the same as the cluster from SLC. A TN isolate did not carry the assigned allele but instead the allele assigned to another cluster, and the inferred cluster was not the same as the cluster from SLC. A FN isolate did not carry the assigned allele but instead the allele assigned to another cluster, and the inferred cluster was the same as the cluster from SLC. The below formulas were used to calculate accuracy, specificity, and sensitivity for each cluster. To summarise the accuracy, sensitivity and specificity for a gene, the individual values calculated for each cluster were averaged. For a gene the macro-average, macro-sensitivity and macro-specificity was the unweighted mean of their respective values when calculating confusion matrixes for each cluster.

$$Accuracy = \frac{TN + TN}{TP + TN + FP + FN}$$

$$Specificity = \frac{TP}{TP + FP}$$

$$Sensitivity = \frac{TP}{TP + FN}$$

1.2 Filtering metrics for selecting a single three gene combination.

A combination of five metrics was used to select a single three gene combination. All metrics were calculated using the definition dataset. The following was calculated: accuracy, sensitivity, specificity, distance separating genes, failed isolates and isolates in non-redundant lineages. The methods for accuracy, specificity and sensitivity are shown in **Methods S1.2**. For the distance separating genes, an inhouse python script calculated the number of nucleotides separating genes of a combination using positions in the GCA_000012045 reference (**Script 3**). For failed isolates, an inhouse python script calculated the percentage of isolates in the definition dataset that did not carry any cluster specific alleles in a gene combination (**Script 2**). For the isolates in non-redundant lineages, an inhouse python script calculated the percentage of isolates in the definition dataset that were assigned a lineage using a single cluster specific allele (**Script 4**). The five metrics were used to progressively filter three gene combinations in the order shown in **Table S1**. Combinations were filtered using the thresholds for each metrics shown in **Table S1**.

2 Supplementary Results

2.1 The seven-gene distribution of *S. aureus*

To select a subset of genomes that represented the species diversity we applied MLST to sub-divide the species. MLST grouped isolates that shared identical alleles for predefined set of seven core loci. MLST analysis of the species dataset (*n=50,481*) defined 1,665 unique STs (**Supplementary Dataset 1**). The frequencies of each ST were visualised and showed MLST divided the species into a few larger STs followed by a large number of smaller STs (**Figure S1**). The ten largest types were ST were assigned to the majority of isolates. Cumulatively, these STs included 68% (34,102/50,481) of isolates. The remaining isolates were assigned to 1,655 STs. A significant number of the smaller STs were small. A total of 90% (1,482/1,665) of these STs were assigned to less than 10 isolates and 962 were singleton STs that had been assigned to a single isolate. In summary, seven-gene MLST divided the species dataset into 1,665 STs. While the ten largest STs included over half the dataset, the overwhelming majority of STs were smaller and formed groups of less than 10 isolates.

2.2 The quality filtered core genome.

A high-quality locus was present and not partially missing in at least 99% of genomes. In total, 1,713 core loci were shown as high quality and remained in the core genome (**Figure S2**). Of the 148 removed loci there was 39 that lacked presence and 109 that were partially missing. The resulting core genome of 1,713 quality filtered loci were used in the following sections for sub-species division.

2.3 Selection of three genes with alleles specific to the 61 Lineages.

To justify selecting a single three gene combination the three gene combinations were filtered with five metrics. These metrics were combined to progressively filter three gene combinations of greater than 99% accuracy (**Table S2, Supplementary Dataset 2**). Firstly, combinations were selected with 100% specificity to 61 lineages. There were 723 combinations with all alleles being lineage specific. Secondly, a minimum distance of nucleotide separated was added. There were 585 combinations with all three genes separated by greater than or equal to 100,000 base pairs. Thirdly, a combination needed to have a cluster specific allele in greater than or equal to 99% of isolates in the definition dataset. Adding this metric reduced the number of combinations to eight. Finally, the combination with the lowest percentage of isolates in non-redundant lineages was chosen. A non-redundant lineage was typable only by an allele from only one of the genes in a three gene combination. The lowest combination for this metric assigned a lineage to 1.93% of the definition dataset using a single allele from the three gene combination. The selected combination was SACOL0451-SACOL1908-SACOL2725 which had 99.71% accuracy, 100% specificity, all genes separated by more than 100,000 base pairs, an allele for assigning a lineage in 99.2% of the definition dataset isolates and described 1.93% of the definition dataset with non-redundant lineages (**Supplementary Dataset 2**).

3 Supplementary Files

3.1 Supplementary Figures

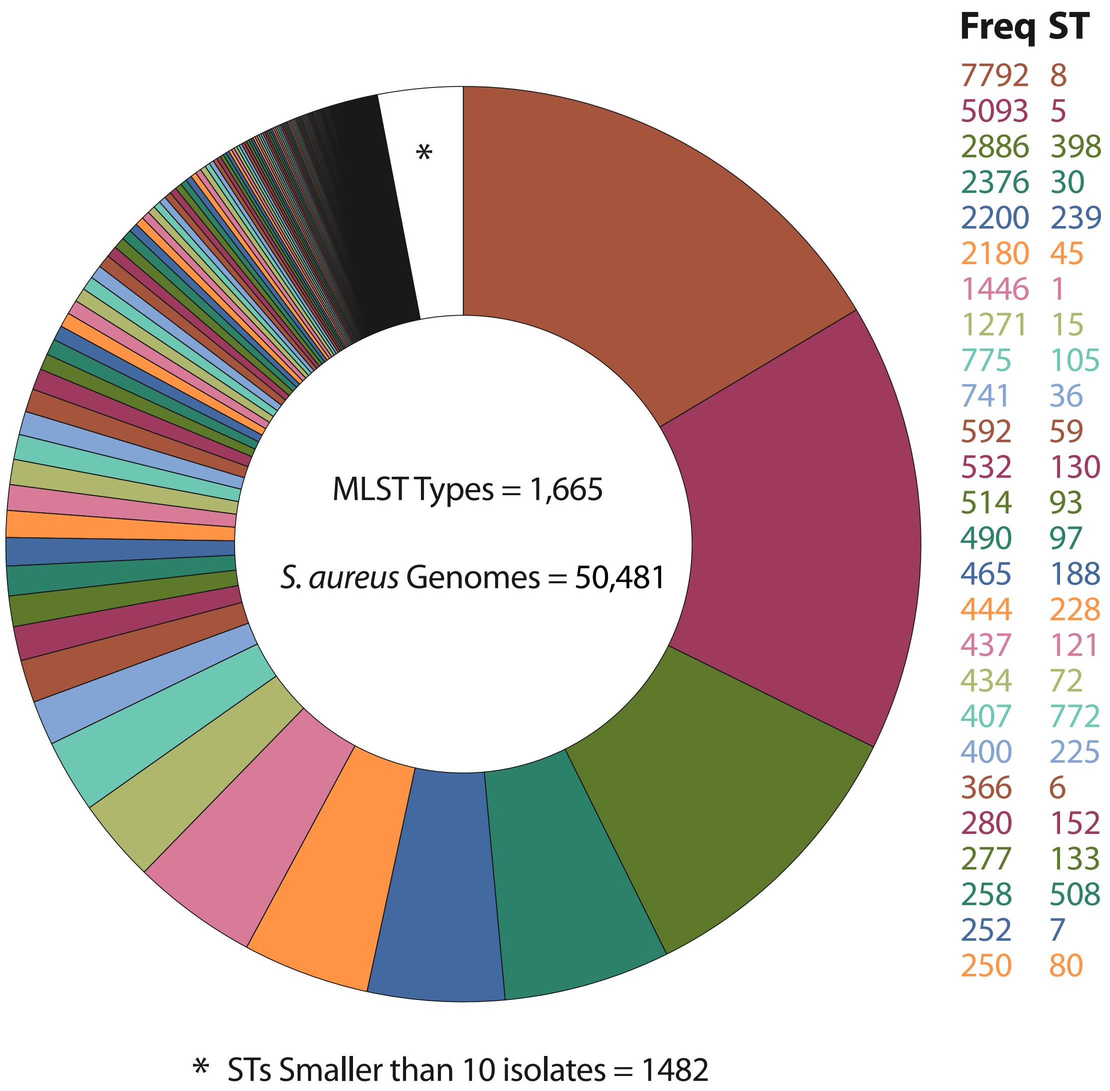

Figure S1. Conventional MLST profiles for the 50,481 *S. aureus* genomes analysed in this study.

The frequencies of 1,665 sequence types (STs) were visualised (in a clockwise direction) from most to least abundant, with ST-8 being dominant in this dataset. Only STs with greater than or equal to 250 isolates were displayed in the key. *STs with fewer than 10 isolates were collapsed into a single white section. Graph was visualised in Prism v9.3.1 (1).

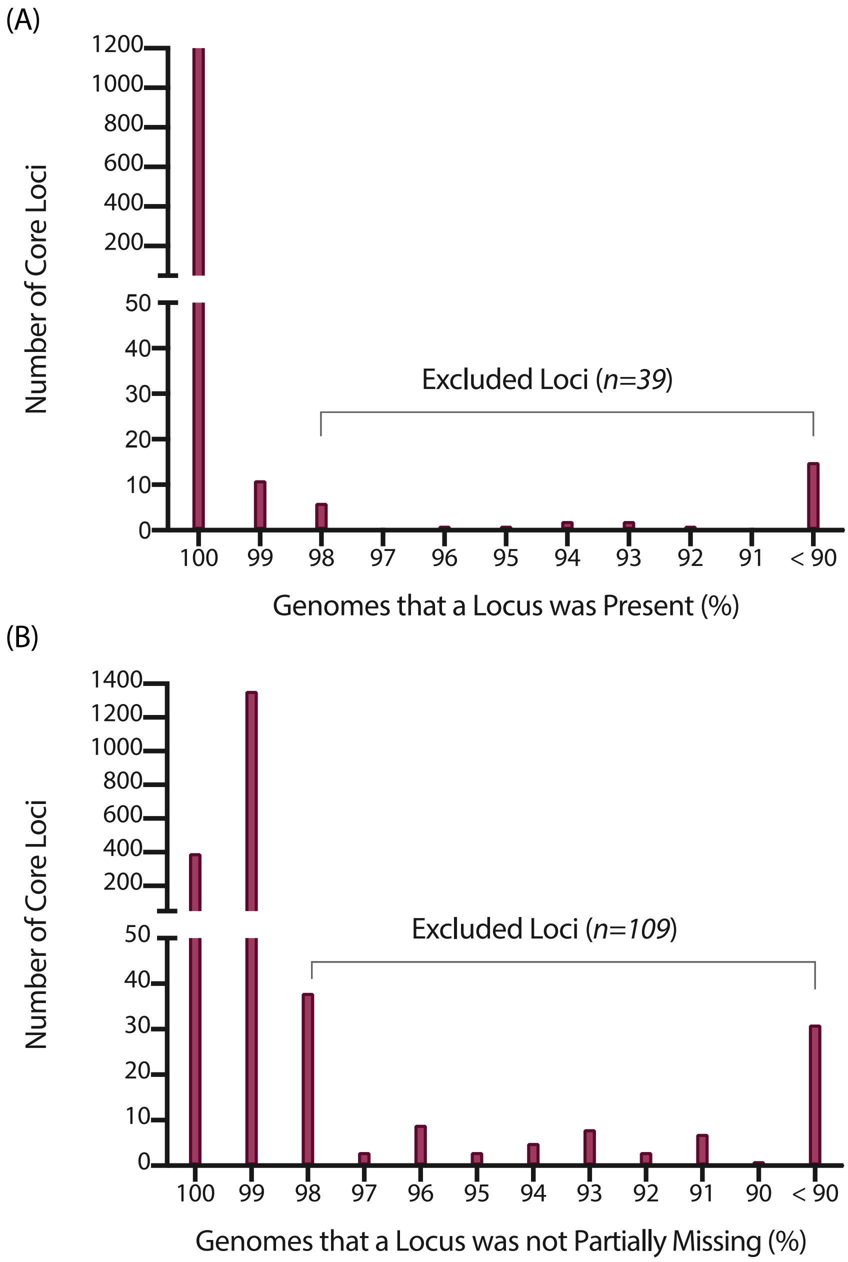

Figure S2. The established core genome was quality filtered.

The genes in the core genome were assessed for absence and completeness in 10,000 *S. aureus isolates*. The MGT pipeline called alleles for all core loci (2). (A) The percentage of genomes a locus was missing. Loci that were missing in more than two percent of genomes were outlined in the grey bracket. (B) The percentage of loci that partially missing. Loci that were negative in more than two percent of isolates were outlined in the grey bracket. Graph was visualised in Prism v9.3.1 (1).

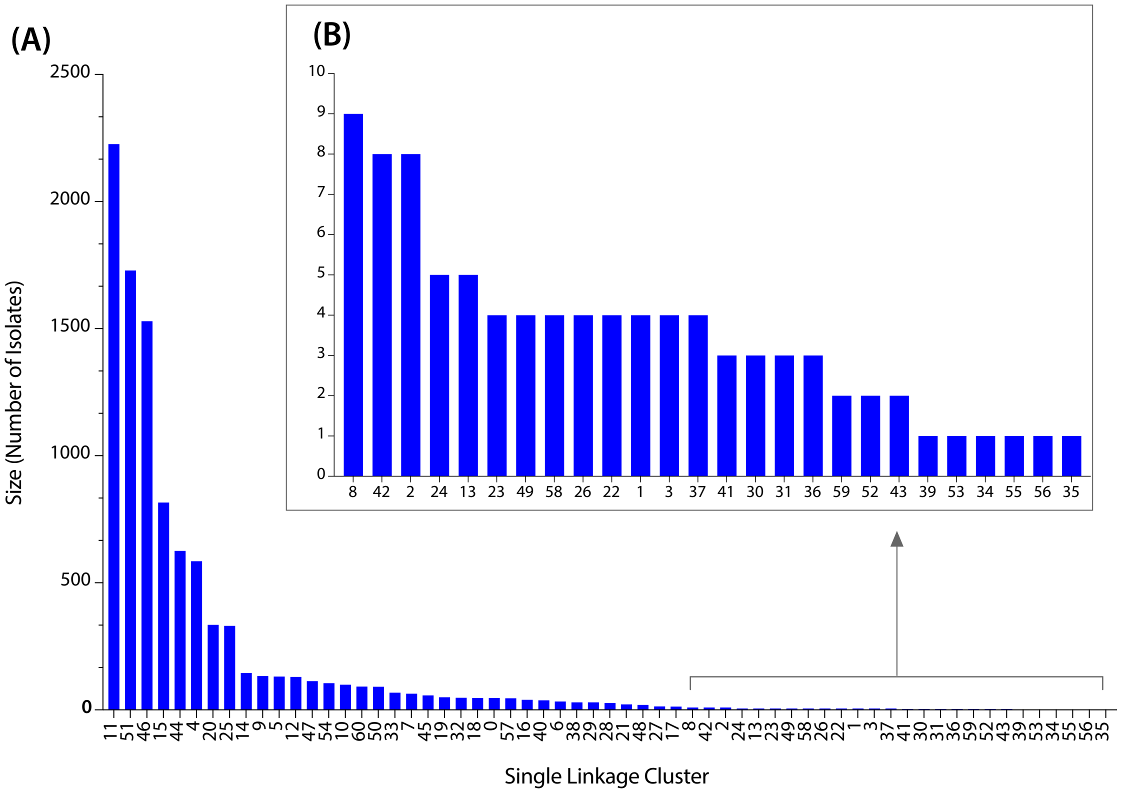

Figure S3. The distribution of 61 cluster sizes that divide the *S. aureus* representative dataset.

The distribution of sizes (number of isolates) for clusters that divided the *S. aureus* representative dataset (n=10,000). (A) The size for all 61 clusters. (B) Sub-plot of 26 clusters that were less than 10 isolates in size. Graph was visualised in Prism v9.3.1 (1).

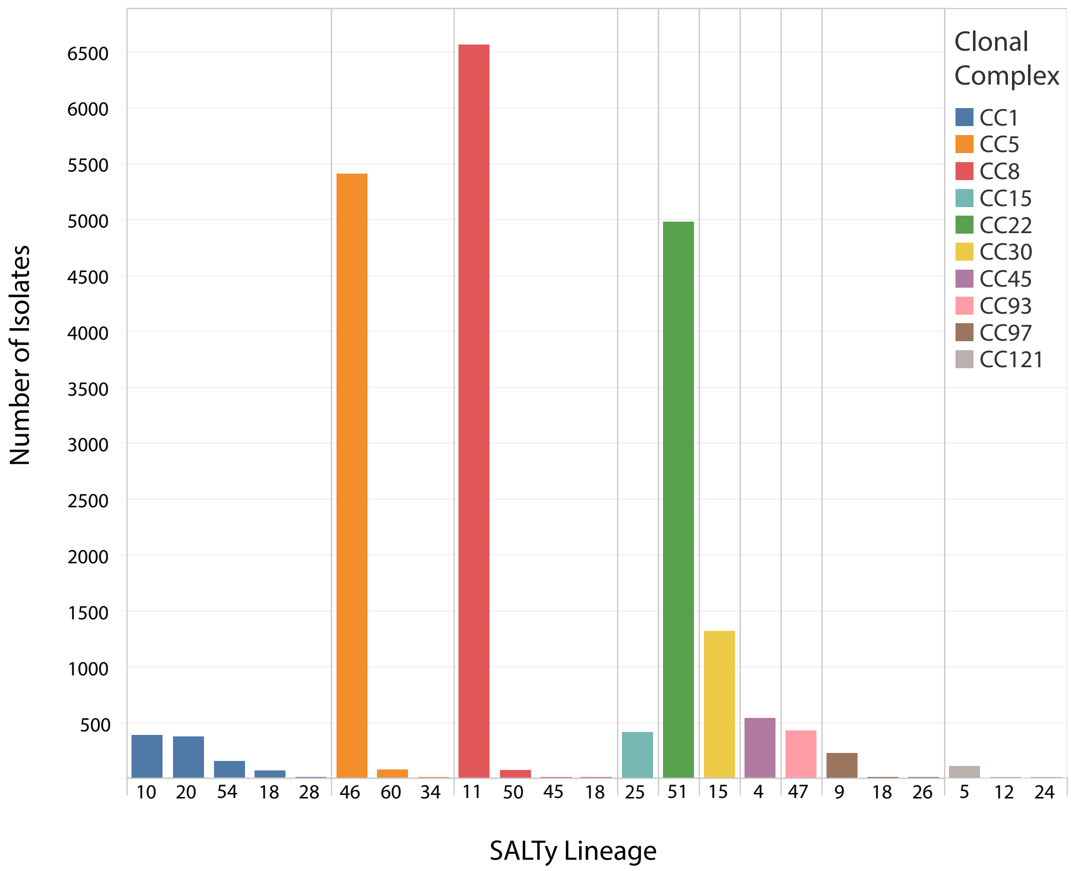

Figure S4. SALTy split half the BIGSdb clonal complexes in into multiple lineages.

BIGSdb had 21,173 *S. aureus* submissions with associated clonal complexing metadata. The SALTy lineage assignments and BIGSdb clonal complex (CCs) were visualised. SALTy divided CCs into multiple lineages (labels underneath). Columns of the graph were grouped and coloured by CC. Within each CC the SALTy lineages were organised in descending number of isolates. Graph was generated in Tableau v2019.4 (1).

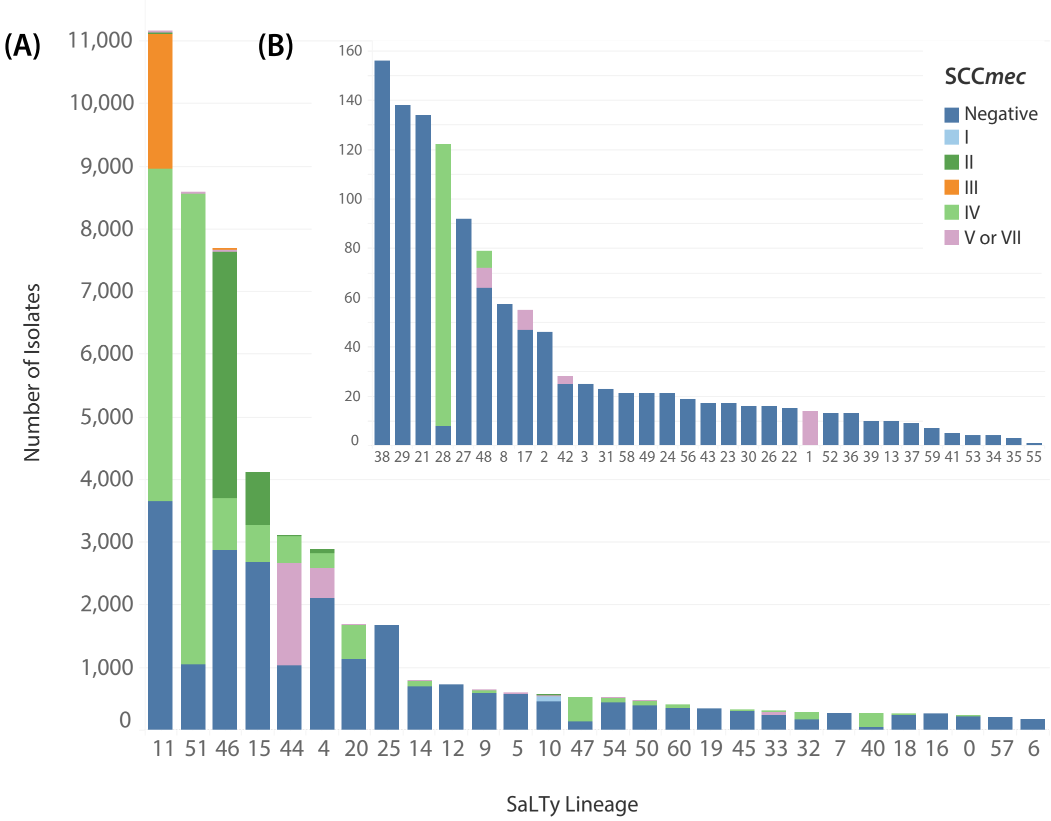

Figure S5. The distribution of SCCmec types for the isolates of each SaLTy lineage.

The SCC*mec* type was predicted in each isolate of the species dataset. Isolates were organised into SaLTy lineages and the lineages were organised by total number of isolates in descending order. Lineages with more than 160 isolates were shown in part A, and lineages with less than or equal to 160 isolates were shown in part B. Isolates were coloured by predicted SCC*mec* type. Non-typable isolates were dark blue and isolates with SCC*mec* type I, II, III, IV and V or VII were coloured as light blue, dark green, orange, light green and purple (respectively). The SCC*mec* type for isolates in each SaLTy lineage was visualised in Tableau (3).

3.2 Supplementary Tables

Table S1. Filtering criteria to select a single combination of three genes.

**Selection Criteria**	**Threshold**	**Three Gene Combinations Passed**
Accuracy (%)	>99	1,726
Specificity (%)	100	723
Distance Separation Loci (bps)	>100,000	585
Failed Isolates (%)	<1	8
Isolates in Non-Redundant Lineages (%)	Lowest	1
**Selected Three Gene Combination**	SACOL0451 - SACOL1908 - SACOL2725	

Table S2. Comparing the average number of antibiotic resistant genes carried by isolates in SALTy lineages and CCs.

Clonal Complex	SALTy Lineage	Mean*	SEM**	Size
CC1	18	6.25	0.4	56
CC1	10	13.09	0.24	384
CC1	20	5.21	0.26	368
CC1	54	3.36	0.22	138
CC1	28	5	N/A	1
CC121	5	5.19	0.2	108
CC121	24	4	0	9
CC121	12	1	0	6
CC125	25	4.49	0.14	411
CC22	51	5.75	0.04	4,984
CC30	15	8.33	0.2	1,317
CC45	4	4.66	0.24	538
CC5	46	11.48	0.1	5,407
CC5	60	3.8	0.28	71
CC5	34	8	0	5
CC8	11	11.27	0.08	6,562
CC8	50	6.37	0.48	67
CC8	45	2.6	1.36	5
CC93	47	3.99	0.08	429
CC97	9	3.26	0.18	225
CC97	26	3.17	0.34	6

3.3 Scripts

The scripts developed to research the findings of this study are openly available at: https://github.com/LanLab/SaLTy/scripts.

Script 1

An in-house python script to calculate the pairwise number of allele differences between cgMLST profiles.

Script 2

An in-house python script to identify alleles of core genes specific to a hierarchical cluster.

Script 3

An inhouse python script to calculate the number of nucleotides separating genes.

Script 4

An inhouse python script to calculate the percentage of isolates assigned a lineage.

4 Supplementary Datasets

Supplementary datasets can be found at the following link: https://figshare.com/articles/dataset/SaLTy_publication_supplementary_datasets/25632786

5 References

1. Hart EB, K. **Prism: Download data from the Oregon prism project**. 2015. Available from: http://github.com/ropensci/prism.

2. Payne M, Kaur S, Wang Q, Hennessy D, Luo L, Octavia S, et al. **Multilevel Genome Typing: genomics-guided scalable resolution typing of microbial pathogens**. Eurosurveillance. 2020;25(20):1900519.

3. Deadorf A. **Tableau (version. 9.1)**. Journal of the Medical Library Association : JMLA. 2016;104(2):182-3.
